# Enantiomeric Aβ peptides inhibit the fluid shear stress response of PIEZO1

**DOI:** 10.1038/s41598-018-32572-2

**Published:** 2018-09-24

**Authors:** Mohammad M. Maneshi, Lynn Ziegler, Frederick Sachs, Susan Z. Hua, Philip A. Gottlieb

**Affiliations:** 1 Department of Physiology and Biophysics, 302 Cary Hall, State University of New York at Buffalo, Buffalo, NY 14214 USA; 2 Department of Mechanical and Aerospace Engineering, 340 Jarvis Hall, State University of New York at Buffalo, Buffalo, New York 14260 USA; 3 Present Address: 745 N Fairbanks, Tarry 7-718, Feinberg School of Medicine, Chicago, IL 60611 USA

## Abstract

Traumatic brain injury (TBI) elevates Abeta (Aβ) peptides in the brain and cerebral spinal fluid. Aβ peptides are amphipathic molecules that can modulate membrane mechanics. Because the mechanosensitive cation channel PIEZO1 is gated by membrane tension and curvature, it prompted us to test the effects of Aβ on PIEZO1. Using precision fluid shear stress as a stimulus, we found that Aβ *monomers* inhibit PIEZO1 at femtomolar to picomolar concentrations. The Aβ oligomers proved much less potent. The effect of Aβs on Piezo gating did not involve peptide-protein interactions since the D and L enantiomers had similar effects. Incubating a fluorescent derivative of Aβ and a fluorescently tagged PIEZO1, we showed that Aβ can colocalize with PIEZO1, suggesting that they both had an affinity for particular regions of the bilayer. To better understand the PIEZO1 inhibitory effects of Aβ, we examined their effect on wound healing. We observed that over-expression of PIEZO1 in HEK293 cells *increased* cell migration velocity ~10-fold, and both enantiomeric Aβ peptides and GsMTx4 independently inhibited migration, demonstrating involvement of PIEZO1 in cell motility. As part of the motility study we examined the correlation of PIEZO1 function with tension in the cytoskeleton using a genetically encoded fluorescent stress probe. Aβ peptides *increased* resting stress in F-actin, and is correlated with Aβ block of PIEZO1-mediated Ca^2+^ influx. Aβ inhibition of PIEZO1 in the absence of *stereospecific* peptide-protein interactions shows that Aβ peptides modulate both cell membrane and cytoskeletal mechanics to control PIEZO1-triggered Ca^2+^ influx.

## Introduction

There is a correlation between traumatic brain injury (TBI), Amyloid β peptide levels (Aβ) and the onset of TBI related diseases^1^. The relationship between Aβ and clinical manifestations of TBI is not understood^1^. Since, the initiating mechanical forces ultimately resulting in TBI appear not to visibly damage cells, a multi-step force signaling mechanism is likely involved, including changes to the cytoskeleton. A potential link in this pathway is the activity of mechanosensitive (MSC) PIEZO channels^2,3^ that respond to stress in the lipid bilayer^4–6^. Since amphipathic drugs including Aβ peptides alter membrane structure^7,8^ and thus membrane mechanics, we examined the effects of Aβs on PIEZO1.

How amphipaths may alter mechanical channels is suggested from studies on bacterial MSCs that respond to global forces applied through bilayer lipids^9,10^ or by lipid perturbations around the channels^11^. The recently published cryoEM structure of mouse Piezo1 in a bilayer dome underscores the connection between lipids and MSCs as an integrated system^12^.

Modulation of Piezo channels by amphipaths can potentially influence a number of cellular responses. For example, the differentiation of mouse neural stem cells into either neurons or glia, requires PIEZO1; inhibition of PIEZO1 by the amphipathic peptide GsMTx4 influences this fate decision^13^. Homeostatic control of epithelial cell number relies on PIEZO1 sensing of cell crowding and cell division^14,15^. PIEZO1 channels are involved in neurite extension and inhibition of PIEZO1 can alter neuronal outgrowth^16^. All these processes are associated with sensing the local mechanical environment^17^.

In this work, we asked whether Aβ can affect PIEZO1. We tested the ability of Aβ peptides to modulate the cellular response to mechanical inputs generated by a precision fluid shear stress system, measuring PIEZO1-mediated Ca^2+^ fluxes. A stable overexpressing PIEZO1 cell line made the response larger and more reliable. Titration of Aβ peptides showed that *monomeric* Aβs in the femtomolar to picomolar range were effective inhibitors, but Aβ *oligomers* were much less potent. PIEZO1 inhibition by D and L enantiomers of Aβs proved equally effective, showing a lack of peptide-protein or other stereospecific interactions. The peptide-channel interaction is likely mediated via bilayer lipids. The inhibitory effects of Aβs were similar to responses to GsMTx4, another amphipathic, stereo-nonspecific inhibitor of PIEZO1^18^ but requiring much higher concentrations. Inhibition of PIEZO1 by Aβs or GsMTx4 almost completely blocked cell migration in a wound healing assay. Because migration requires a pliable cytoskeleton, we measured the effect of Aβs on cytoskeletal stresses using a genetically encoded optical force probes embedded in actin^19^. The inhibition of PIEZO1 by Aβ and the resulting block of Ca^2+^ influx was accompanied by significantly *increased* actin tension. PIEZO1 activity is causally linked to cell motility and cytoskeletal reorganization.

## Results

### Fluid shear stress assay

We first created a cell line that expressed a fluorescently tagged version of human PIEZO1 called hPIEZO1-1591-EGFP^5^. The fusion protein’s encoded internal GFP and N-terminal His tag did not significantly affect the channel properties^5^. The cDNA was integrated into the genome of HEK293T cells using a lentivirus vector. The single channel conductance of this stably overexpressing PIEZO1 cell line (hP1-CL) was indistinguishable from that of hPIEZO1 in transiently transfected HEK293T cells (Supplementary Fig. 1).

### Aβ peptide inhibition of PIEZO1

**Figure 1:**
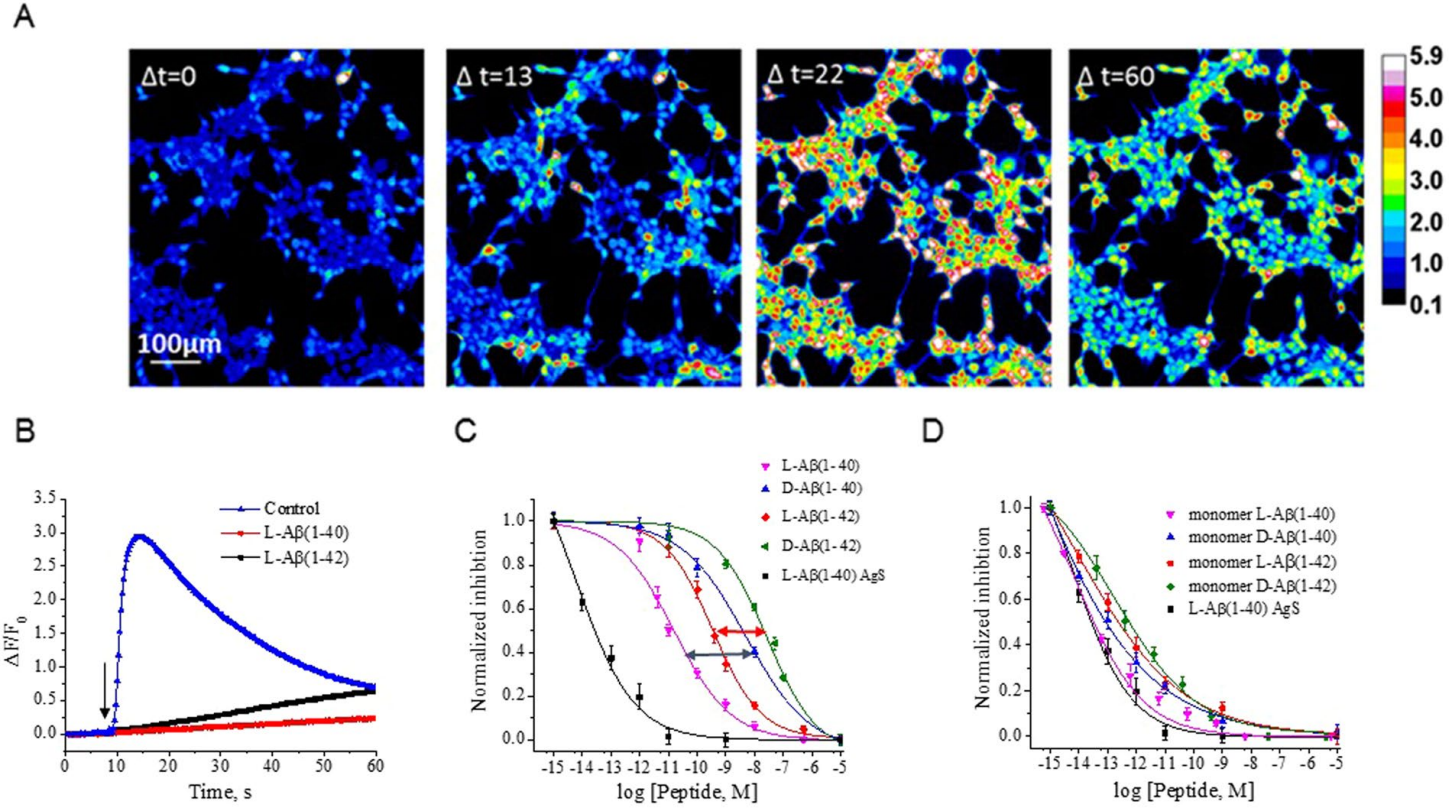
Aβ peptides inhibit shear stress-induced elevation of [Ca^2+^]_i_ in a cell line stably overexpressing PIEZO1. (Panel A) hP1-CL clonal cell line was grown in microfluidic chambers, loaded with Fluo-4 AM, and stimulated with 10 ms pulses of 15 dynes/cm^2^. Changes in fluorescence were monitored by a CCD camera at 1 Hz. Shown are intensity changes at four indicated time points. The maximum response is observed at ∆t = 22 s, long after termination of the stimulus. (Panel B). The L forms of Aβ(1-40) or (1-42) prepared by Peptide Method I at 10 µM to hP1-CL cells inhibited the shear-induced elevation of [Ca^2+^]_i_ response (4 independent experiments with SEM). (Panel C). PIEZO1 inhibitory potencies of the enantiomeric Aβ peptides (prepared by Peptide Method I) and of the commercially available monomeric peptide AgS, called Aggresure L- Aβ(1-40). The K_i_ (half-maximal concentration for inhibition) was determined based on the peak response plotted as a function of concentration. Each concentration is an average of 4 independent experiments with SEM. We observed K_i_ differences between the D and L forms of each peptide (diastereomeric pairs are identified with horizontal double-headed arrows). (Panel D) Monomeric Aβ peptides prepared by Peptide Method II were tested. The monomeric L-Aβ(1-40) was nearly identical to AgS in its ability to inhibit PIEZO1 function with a K_i_ of ~50 fM (4 independent experiments with SEM).

hP1-CL cells were grown on fibronectin-coated microfluidic chambers (Supplementary Fig. 2) and loaded with the calcium indicator, Fluo4-AM and subjected to single square shear pulses^20^; 10 ms duration at ~15 dynes/cm^2^. The resulting robust increase in [Ca^2+^]_i_ (Fig. 1A,B, blue triangles) reached a maximum about ∆t = 22 s, followed by a loss of fluorescence indicating adaptation or inactivation (∆t = 60 s). The time course for fluorescence decay followed a single exponential with τ = 132 ± 10 s (6 traces from 4 independent experiments with SEM). The long delay between the stimulus and the peak response probably reflects amplification by CICR (Ca^2+^ induced Ca^2+^ release). Control HEK293T cells showed a minimal PIEZO1 response (Supplementary Fig. 3A, black squares) at the same magnitude of shear stress (Supplementary Fig. 3A represents the response of 4 independent experiments). Cytoskeletal disruption of hP1-CL cells with either cytochalasin D (Supplementary Fig. 3A, green triangles) or colchicine (Supplementary Fig. 3A, dark blue triangles) produced cells unresponsive to fluid shear, suggesting a requirement of the PIEZO1 shear response for cytoskeletal integrity coupled to the bilayer. This is similar to our observation that mouse PIEZO1 whole cell currents required an intact cytoskeleton to respond to cell indentation^21^.

We tested two Aβ peptides and found that both inhibited the shear response (Fig. 1B). Incubation of hP1-CL with 10 µM L-Aβ(1-40) or L-Aβ(1-42) peptides (see Peptide Method I in Methods section below) inhibited the fluid shear response (Fig. 1B, red circles and black squares, represents 4 independent experiments). Neither the addition of scrambled L-Aβ(1-40) peptide to hP1-CL cells nor expression of Amyloid Precursor Protein (APP)-YFP in hP1-CL had an inhibitory effect on the shear response (Supplementary Fig. 4 left and middle panel). The right panel (Supplementary Fig. 4) shows the average response of 4 independent experiments with SEM.

As a control, we examined the responses to the known inhibitor, D-GsMTx4^18^. At 5 µM, we observed complete inhibition of the response in the shear assay (Supplementary Fig. 3A, light blue diamonds, 3 independent experiments with SEM). Supplementary Fig. 3B shows that responses to shear stress were similar in the stably transduced cell line and in transiently transfected cells, whereas cells expressing only mCherry produced no response (Supplementary Fig. 3B). Suppressing PIEZO1 expression with miRNAs also inhibited the response, but scrambled miRNA sequences had no effect (Supplementary Fig. 3B). These data are summarized in Panel B (Supplementary Fig. 3) and is an average of 3 independent experiments with SEM.

We examined whether the Aβ could inhibit PIEZO1 currents in the patch. We formed outside/out patches from the hP1 cell line and tested whether 10 µM of Aβ (1-40) or Aβ (1-42) (Peptide Method I) inhibited channel activity, a concentration that blocked the shear response. Surprisingly, neither peptide inhibited outside/out patch currents (Supplementary Fig. 5, represents 3 independent experiments).

Peptide binding specificity to the channel was assessed using the L and D enantiomers of Aβ(1-40) and Aβ(1-42), (c.f.^22^). For all these experiments, each data point is an average of 4 independent experiments with SEM. The ratio of inhibitory constants, K_i_, between of the L and D Aβ peptides was ~300-fold for Aβ(1-40) (Fig. 1C, blue double arrow) and ~70-fold for Aβ(1-42) (Fig. 1C, red double arrow). Using the scrambled peptide as an estimate of non-specific binding, at least a > 10,000-fold difference between L and D forms was expected because the D-form will be incapable of specific interactions. Since we observed only a 300-fold difference, it indicated a mechanism that does not involve peptide-channel interactions. The monomeric peptide AgS (AggreSure L-Aβ(1-40); Fig. 1C, black squares**)** inhibited PIEZO1 with a K_i_ about two orders of magnitude lower (~30 fM), and indicated that the sensitivity of enantiomeric peptides is related to state of the peptide (i.e. aggregate versus monomer).

### PIEZO1 inhibition by monomeric peptides

We prepared monomers of four Aβ peptides (see Peptide-Method II) and titrated their inhibitory effects on the shear response of hP1-CL cells (Fig. 1D, 4 independent experiments with SEM indicated for each concentration). The difference between the K_i_ values of AgS peptide (~30 fM) and monomerized L-Aβ(1-40) (~52 fM) was negligible (Table 1). The K_i_s for all the monomeric peptides were in the fM to sub-pM range, with K_i_ values for enantiomeric pairs of Aβ(1-40) and Aβ(1-42) differing by only 2- to-3-fold (Table 1). These results are consistent with the assumption that peptides prepared by method I differed in oligomeric state (or conformation) from AgS in a way that reduced PIEZO1 inhibitory potency by 3–6 orders of magnitude. The similar efficacy of the D and L peptides suggests that they only interact with PIEZO1 through non-contact (lipidic) mechanisms.

**Table 1:** Calculated K_i_ for inhibition of hPIEZO1-mediated, shear stress-induced [Ca^2+^]i increase by enantiomeric Aβ peptides prepared by Method 1 (monomer, rows 2–5) or by Method 2 (oligomer, rows 6–9), and for the commercially available monomeric peptide, AggreSure 40 (row 1).

Peptide	K_i_ [M]	K_err_	n	n_err_	Adjusted R^2^
AggreSure 40	31.2 e-15	6.8 e-15	0.50	0.06	0.987
L40 monomer	51.5 e-15	9.9 e-15	0.49	0.05	0.997
D40 monomer	112.6 e-15	14.3 e-15	0.34	0.02	0.998
L42 monomer	261.5 e-15	19.7 e-15	0.40	0.04	0.986
D42 monomer	725.5 e-15	98.8 e-15	0.37	0.09	0.990
L40	15.8 e-12	2.58 e-12	0.48	0.05	0.990
D40	4.9 e-9	683e-12	0.34	0.03	0.998
L42	345 e-12	49.11 e-12	0.52	0.04	0.995
D42	26.6 e-9	6.7 e-9	0.49	0.05	0.989

We were unable to washout the channel inhibition within 20 min or by increasing the shear stress to 25 dynes/cm^2^. In conventional solution kinetics, the upper limit for the association rate constant (k_a_) is usually considered to be ~10^8^ M^−1^ s^−1^, a diffusion-controlled limit. The dissociation rate constant (k_d_) therefore should be less than 10^−4^ s^−1^, and the time constant for achieving binding equilibrium at a peptide concentration = K_i_, will be on the order of 10^4^ s (a few hours). If the dissociation constant is 100 fM, the time constant would be about 10^5^ s (a day, or so). The fact that we achieved equilibrium in much shorter times suggests that the local membrane concentration was much higher than in solution.

Titration of the Aβ response showed a negatively cooperative Hill coefficient <1 suggesting that higher concentrations buffer the monomers into less effective oligomers. In contrast to Aβ inhibition, GsMTx4, had a much higher K_i_ of ~250 nM^23,24^ with a Hill coefficient of ~ 1.9 (Supplementary Fig. 3C**)**, and channel activity was readily restored by GsMTx4 washout (data not shown).

### Co-localization of peptide and channel

**Figure 2:**
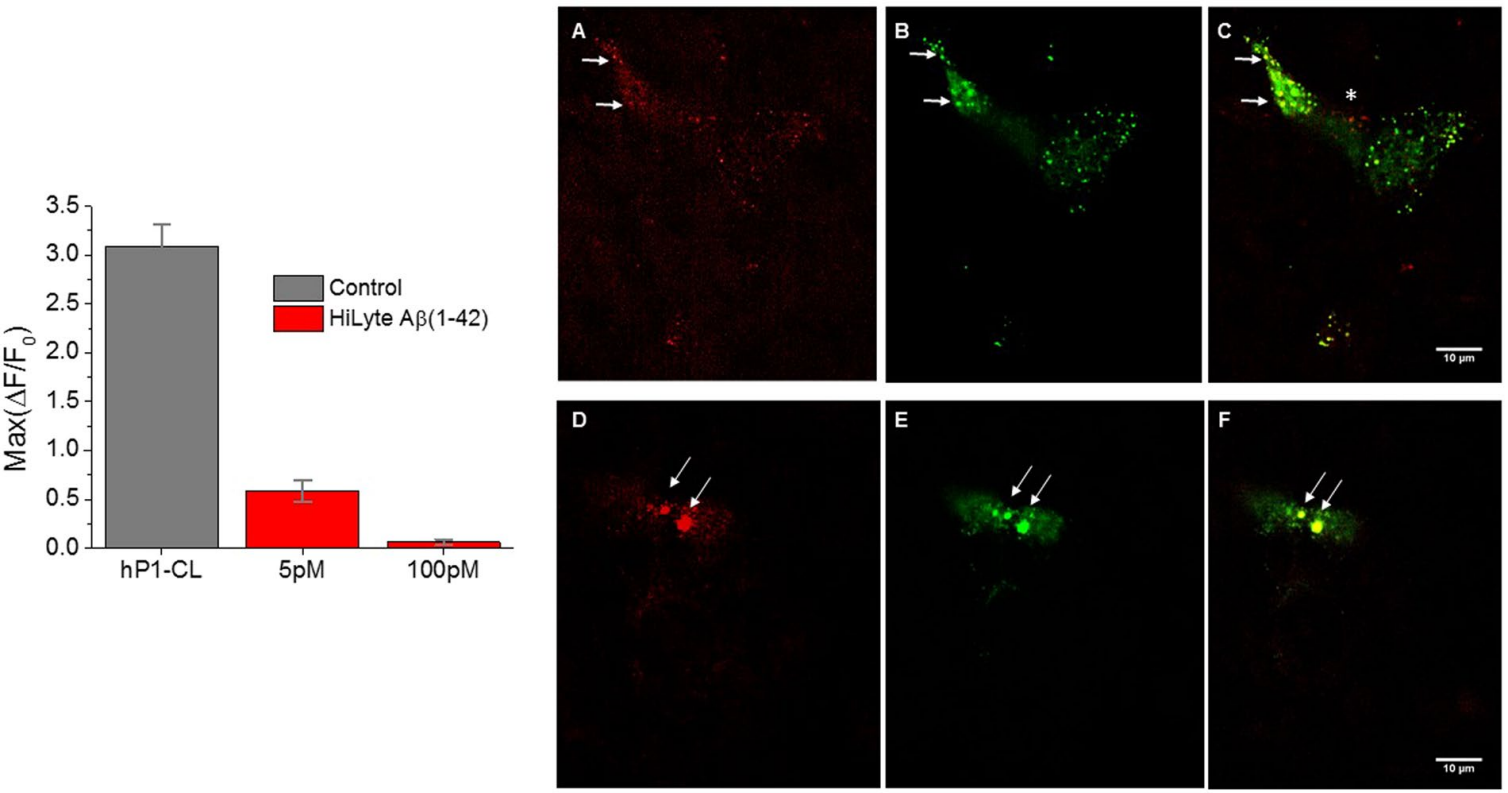
Aβ peptide co-localizes with PIEZO1. L-Aβ(1-42) fluorescently labeled at the N-terminus with HiLyte (555) was purchased (Anaspec) and monomerized. Left-The peptide inhibited the shear stress response of the hPIEZO1 cell line at 5 pM and 100 pM (4 independent experiments with SEM). Right (Panels A–F) –Confocal z stack images of colocalized HiLyte Aβ(1-42) and PIEZO cell line. Panels A through C are images near the bottom of the cell (z = 5/60). Panel A is the red channel, Panel B is the green channel and Panel C is the overlay. Examples of overlap (yellow) are indicated by arrows. Panels D through F are images near the top of the cell z = 49/60. Panel D is the red channel, Panel E is the green channel, and Panel F is the overlay. Note the rare red sites indicating unassociated Aβ peptide, indicated by an asterisk.

Monomerized red fluorescent Aβ(1-42) peptide at 100 pM completely inhibited the PIEZO1-mediated Ca^2+^ response to shear stress **(**Fig. 2-Left Panel, 4 independent experiments with SEM). We incubated 100 pM of fluorescent Aβ with fluorescent hP1-CL cells for 10 min and investigated possible PIEZO1 co-localization with the peptide (Fig. 2**-**Right Panels marked A–F) using Structured Illumination Microscopy (SIM). Panels A–C are images near the bottom of the cell. Panel A is the peptide (red) and Panel B is the PIEZO1 channel (green). Panel C is the overlay of the red and green fluorescent channels showing yellow puncta where there is overlap indicating co-localization (indicated by arrows). Note that not all channels are associated with Aβ (1-42) peptide. Panels D–F are images near the top of the cell which also show co-localization of the peptide and PIEZO1 (indicated by arrows). Piezo proteins are known to segregate into spatial domains^25,26^ in the absence of Aβs, and the altered environment of the channels may favor Aβ association.

### Aβ peptides affect PIEZO1-mediated cell migration

PIEZO1 channels are involved in neurite extension^16^ and other types of cell motility. This led us to test the effects of Aβ peptides on hP1-CL motility in a wound healing assay. Cells were grown to confluence on glass coverslips half-coated with PDMS (Polydimethylsiloxane). The PDMS layer was then peeled off allowing us to accurately measure the collective cell migration rates across the newly exposed glass surface.

**Figure 3:**
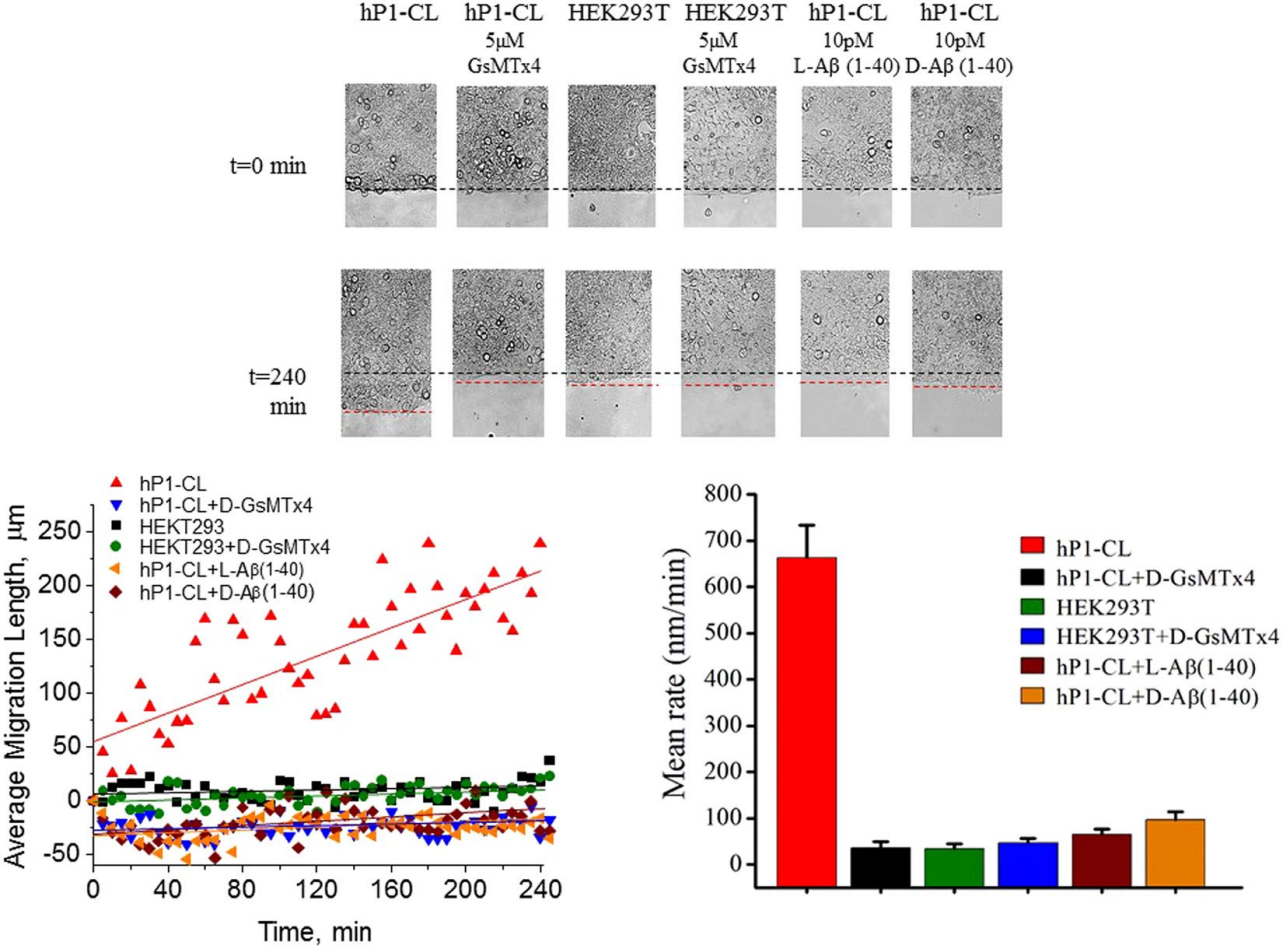
Aβ peptides inhibit migration of hP1-CL cells. Cell migration was measured as cell movement on glass cover slips in polystyrene dishes at 37 °C and 5% CO_2_. A PDMS barrier to cell growth was created on the cover slip. When cells reached confluency, the barrier was peeled off. Images of the resulting, initially sharp confluent cell boundary were captured at 0.003 Hz over 5 hours. Top panel shows initial (t = 0 min, black dotted lines) and final time points (t = 240 min, red dotted lines) of cell migration. hP1-CL cell migration was blocked by 5 µM D-GsMTx4, as well as 10 pM concentrations of L and D Aβ(1-40). Bottom left panel summarizes the mean cell migration distances as a function of time. Bottom right panel is the mean migration rate of hP1-CL cells in presence and absence of 5 µM D-GsMTx4, and of 10 pM of the L or D forms of Aβ(1-40). Three independent experiments were averaged (SEM).

Surprisingly, hP1-CL cells migrated ~10-fold faster than native HEK293T cells (Fig. 3 Top panel) showing the involvement of PIEZO1 channels in cell sheet movement. Addition of either D-GsMTx4 (5 µM) or enantiomeric forms of monomeric Aβ(1-40) (10 pM) slowed cell migration by over 10-fold, consistent with PIEZO1 involvement in cell movement (Fig. 3 Bottom left panel). Figure 3 Bottom right panel shows the average velocity for 3 independent experiments with SEM.

### Aβ peptides are involved in cytoskeletal remodeling

**Figure 4:**
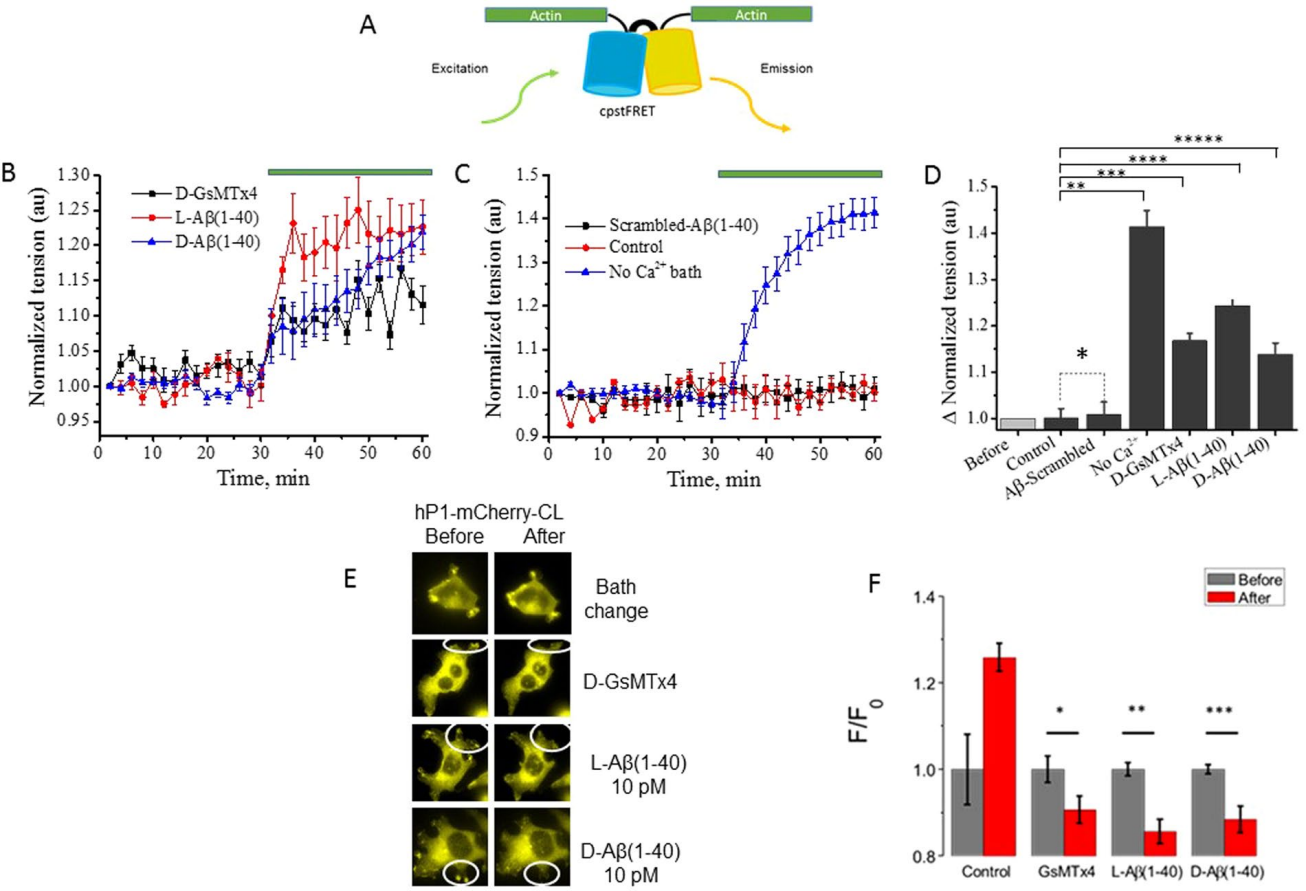
D**-**GsMTx4 and the enantiomeric Aβ(1-40) peptides inhibit Ca^2+^ entry, and increase cytoskeleton stress in hP1-mCherry-CL cells. hP1-mCherry-CL cells were transiently transfected with cDNA encoding the actin force probe to monitor peptide-induced changes to the cell’s cytoskeleton. Panel A shows the force probe, with the cpstFRET pair linked to actin at both its N- and C-termini^19^. Panel B shows significant changes in actin stress upon exposure to amphipathic peptides. Each data point is the average of 15 cells from 3 different experiments (with SD). A baseline response is established in the first 30 minutes (37 °C and 5% CO_2_). Addition (green bar) of D-GsMTx4 (5 uM, black squares) or monomeric enantiomeric Aβ(1-40) peptides (10 pM; L, red circles; D, blue triangles) elicits immediate increases in actin stress. Panel C summary data shows no change in resting actin stress upon addition of scrambled Aβ(1-40) peptide (10 pM, black squares) or by the control shear stress associated with solution change (red circles). Bath removal of extracellular Ca^2+^ elicits the largest effect (40%, blue triangles). Data points are 15 cells from 3 independent experiments with SD. Panel D summarizes Aβ responses at the 60 min time point (paired t-test). The scrambled Aβ peptide had no effect on the cells (*P = 0.813), whereas bath Ca^2+^ removal increased actin stress (**P < 0.0001). Enantiomeric Aβ(1-40) peptides and D-GsMTx4 all increased actin stress (paired t-test *** and ****P < 0.0001; *****P < 0.0004). Panel E presents actin force probe images of hP1-mCherry-CL cells highlighting lamellipodia. Addition of D-GsMTx4 or the enantiomeric Aβ peptides reduced actin-force probe FRET emissions (as shown by loss of fluorescent signal in the circled regions). Panel F shows changes to fluorescence after retraction of actin from lamellipodia. Data normalized using the fluorescent intensity before the addition of peptide (F_0_). The change in intensity is F/F_0_ where F is the final measurement in the series. The data average 20 lamellipodia from 4 cells (with SEM). Before and after intensities were analyzed by paired t-test (*P < 0.04; **P < 0.0001; ***P < 0.01). Note that the control *increased* fluorescent intensity.

Cell movement in the presence of PIEZO1 channels appears to rely on cytoskeletal integrity and plasticity, consistent with the ability of cytoskeletal disrupting agents to prevent PIEZO1 activation (Supplementary Fig. 4A). We examined forces within the cytoskeleton during channel inhibition. By transient transfection, we introduced a genetically encoded optical probe of actin stress (cpst-FRET) into stable hP1-mCherry-CL cells^19^ (Fig. 4A), resulting in probe expression into F-actin. The fluorescence spectrum of hP1-mCherry-1591 does not significantly overlap with the spectrum of the actin force sensor. For each experiments described below, we analyzed and averaged (with SD) 15 cells from 3 different experiments. After establishing baseline actin stress for 30 min, addition of the peptides resulted in an increase in actin stress (Fig. 4B). 10 pM of monomeric L-Aβ(1-40) (red circles) appeared slightly more effective than D-Aβ (1-40) (blue triangles) or 5 µM D-GsMTx4 (black squares) in increasing actin-associated tension. Figure 4C shows that the fluid shear stress associated with a simple bath exchange had no effect on actin stress (red circles). 10 pM of monomerized scrambled Aβ peptide (black squares) also had no effect on actin stress. However, removal of Ca^2+^ from the extracellular bath greatly *increased* the resting actin stress (blue triangles). The steady state changes in actin stress (at t = 60 min) are summarized in Fig. 4D.

**Figure 5:**
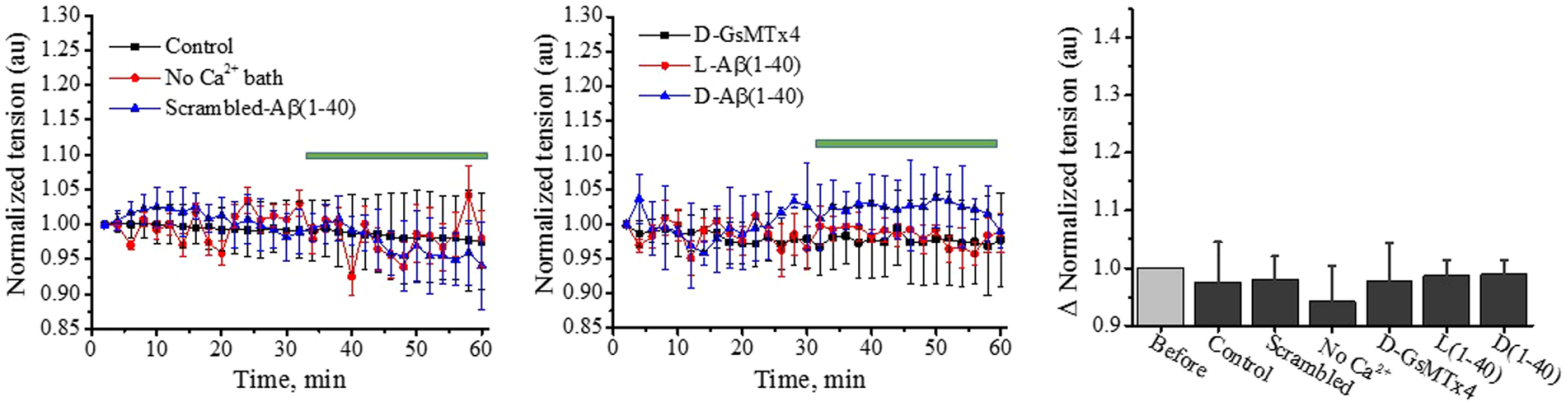
D-GsMTx4 and enantiomeric Aβ(1-40) peptides did not alter actin stress of unmodified HEK293T cells expressing the actin force probe. (Panel A) HEK293T cells were monitored for 30 min to establish a baseline. Upon removal of bath Ca^2+^, addition of scrambled Aβ peptide, or control bath solution exchange (blue bar), the actin stress probe showed no change in FRET. (Panel B) Addition of D-GsMTx4 (5 µM) or the enantiomeric Aβ(1-40) peptides (10 pM) similarly showed no effect on actin stress in these cells. Panel C summarizing the response of control HEK293T cells (15 individual cells from 3 different experiments with SD) shows that actin stress is unaffected by D-GsMTx4 or enantiomeric Aβ peptides in cells lacking PIEZO1 overexpression.

Interestingly, FRET images of hP1-mCherry-CL cells show lamellipodia containing the actin probe (Fig. 4E). Addition of D-GsMTx4 or Aβ (1-40) enantiomers led to a drop of F-actin tension and its retraction from the lamellipodia (Fig. 4E,F). Our results support a model where Ca^2+^ influx through PIEZO1 channels maintains the cytoskeletal plasticity required for cell migration. Control experiments with native HEK293T cells revealed no effects on actin tension by bath Ca^2+^ removal, D-GsMTx-4, or Aβ peptides (Fig. 5).

## Discussion

An important advantage to the shear stress approach, relative to techniques such as patch clamp^27^, is the ability to measure PIEZO1 channel function without altering cellular integrity. We know that the channel’s environment clearly affects the channel’s response to mechanical stress^21^. Assays intended to mimic the *in situ* situation must leave the environment minimally altered. We have shown that monomeric Aβ peptides can block PIEZO1 activity at fM concentrations, and that other forms of the peptides (i.e. aggregated) are less potent. Using enantiomers we demonstrated that the Aβ effects are dominated by long range effects that are not stereospecific similar to the observations seen with GsMTx4^22^.

We examined the electrophysiological effects of Aβs using patch clamp. In control cells, channel activity in response to stretch was obvious^18,21^. We could not observe PIEZO1 inhibition by Aβ peptides at concentrations that inhibited the response to fluid shear stress. The inability to observe patch current correlations with the extreme sensitivity of the shear stress assay is a warning that extrapolation of patch results does not always apply to *in situ* situations. The stresses in patch experiments^28^ may alter the binding of the Aβs or the ability of the peptide perturbations to reach the channels.

How may Aβs affect channel function? Mouse PIEZO1 gate by local bilayer tension^5,6^. In neurons, PIEZO1 activity appears concentrated in cholesterol-rich lipid raft domains containing Stomatin-like 3 protein (STOML3)^26,29^. These membrane nanostructures can redirect the mechanical forces that modulate MSCs^26,30^. The insertion of Aβ peptides into a confined lipid domain compresses^31^ the surrounding lipid and channels. Alternatively, Aβ disrupts the boundary lipids of PIEZO1^11^, as suggested by cryoEM^12^. Perhaps Aβs perturb these structural lipids rendering the channel inactive. Aβ peptides are known to concentrate in lipid rafts^32,33^. The extremely low concentration of monomeric peptide needed to inhibit channel function (fM) may reflect a partition coefficient that concentrates monomeric peptides in constrained domains containing PIEZO1.

Another possibility is that Aβ affects PIEZO1 activity indirectly. Activation of PIEZO1 channels may result from forces created through the cytoskeleton. Cells generate traction forces by interactions with the extracellular matrix (ECM) and can use integrin rich focal adhesion regions to transmit tension to the bilayer activating PIEZO1 channels^34^. The connection between PIEZO1 (Fam38a) and integrins was demonstrated prior to the discovery of PIEZO1’s mechanical channel properties^35^ and activation of PIEZO1 channels through the ECM was shown by Poole *et al*.^26^. The disruption of PIEZO1 activity by Aβ would be attributed to inhibition of any step along a signaling pathway.

Aβ peptides vary considerably in their structure and oligomeric states^36^. Moreover, opinions vary regarding toxicity of the various peptide forms, ranging from soluble dimer/trimers to fibers^37^. Recent work suggests that Aβ monomers are random coils^38^.

Although Aβ peptides are often associated with Alzheimer’s disease, monomeric Aβ peptides at low concentration can exert positive physiological effects on synaptic plasticity and neuronal survival^39,40^. Giuffrida *et al*.^41^ reported the interesting observation that monomeric Aβ (1–42) is neuroprotective, preventing trophic deprivation in developing neurons and protecting mature neurons against excitotoxic death at low concentrations. We have shown the connection between PIEZO1-mediated Ca^2+^ influx and cytoskeleton remodeling and cell mechanics (Fig. 4). The elevation in Aβ peptide concentration following head trauma might be intended to limit the PIEZO1 response to mechanical activation. Consistent with our results on the loss of efficacy with oligomer formation, Giuffrida *et al*.^41^ suggested that oligomerization decreases the concentration of monomers, and may explain a negative Hill coefficient (Table 1). These results also suggest that other amphipathic molecules may also exhibit environmentally sensitive affinities as a function of bilayer tension^31^.

The coupling of PIEZO activity to motility is clear from the effects of over-expression in the HEK293T cells, and inhibition by Aβ peptides. Collective cell migration is related to tissue remodeling events involved in wound healing and cancer^42^, and is distinct from anchorage-independent (“amoeboid”) cell migration observed in the setting of FAM38a (PIEZO1) depletion^35,43^. Linking the flux of Ca^2+^ through PIEZO1 to cytoskeletal changes^34^ allows for rapid and coordinated cell migration. The ubiquitous expression of Aβ peptide^44^ raises the question of how it may influence mechanical responses throughout the organism.

## Methods

### PIEZO1 cell lines

#### Vector hP1-1591-mCherry

The vector hP1-1591-EGFP-mCherry with an N-terminal his tag was amplified using a BamH1 forward primer and EcoR1 reverse primer. The gel purified PCR product was used in an InFusion reaction with pBabe puro vector DNA that had been cut with BamH1 and EcoR1. The resulting construct, pBabePuro-NHis- hP1-1591-mCherry was treated with restriction enzymes MluI and XhoI. A PIEZO1 cDNA fragment containing inserted EGFP at amino acid residue 1591 and with MluI and XhoI ends was inserted by ligation (pBabePuro-NHis-hP1-1591-EGFP). Integrity of changes was confirmed by DNA sequencing.

### Primers

InfHP1BabeBamHisF – GGCGCCGGCCGGATCCTCAGCCACCATGCACCATCATCATC InfHP1BabeR1rev – CACCGGTACTGAATTCCTACTACTCCTTCTCACGAGTCC.

#### Generation of stable cell line

To generate viral particles, AmphoPak cells in 30 mm dishes were grown to 25% confluency and transfected with DNA (1 µg per dish) using Mirus transfection reagent according to manufacturer’s recommendation. After 2 days, the media was collected and filtered through a 45 micron filter.

1 ml freshly harvested virus plus 2 µg/ml polybrene were added to adherent HEK293T cells grown to 25–50% confluency in 30 mm dishes. Fresh medium was added after 3 hrs, and the cells were allowed to incubate overnight. Lentiviral infection was repeated after 24 hours. The twice infected cells were allowed to incubate for an additional 24 hours, and subjected to puromycin selection (Sigma) at four concentrations between 0.25 to 4.0 ug/ml. Single colonies were generated from cells grown at 2.8 µg/ml puromycin. The resulting clonal hP1-CL cell line was used in subsequent experiments.

#### Shear stress assay

The microfluidic chambers had glass coverslip bottoms coated with human fibronectin (Invitrogen)^20^ and fluid flow guided by PDMS channels^20^. Cells were cultured in the chamber for 3 days, with daily medium changes. On the experimental day, chambers were rinsed with isotonic solution (75 mM NaCl, 5 mM KCl, 2 mM MgCl_2_, 1 mM CaCl_2_ and 10 mM HEPES, adjusted to 320 mOsm with mannitol). A stock of Fluo-4 AM was diluted to 5 µM in isotonic saline and incubated with cells for 10–30 min at 37C°. Chambers were then rinsed twice with isotonic solution and incubated for 8 minutes to complete Fluo-4 AM de-esterification. A single fluid shear stress pulse of ~15 dynes/cm^2^ for 8–10 ms was applied to the chamber using a high-speed pressure servo (HSPC-1 from ALA). Peptide stocks were kept at 4 °C until used, dissolved to desired concentrations, and immediately added to cells. Each chamber had a minimum of 15 cells.

Images of the cellular response to shear stress were captured by an EM-CCD camera (C9100 model, Hamamatsu) at a nominal rate of 1 Hz. To quantify the response, the average intensity in the field of view was background subtracted^45^. Image intensity was adjusted for the rate of bleaching. Each condition was repeated in four separate chambers, and the results averaged. The responses were normalized to the control experiments, and the inhibition was calculated as previously described^46^. The data points were fit by a Hill plot with the equation:

1 $$y=\frac{{V}_{max}\,\ast \,{x}^{n}}{({k}^{n}+{x}^{n})}$$

where *Vmax* is the maximum response, *n* is the Hill coefficient, and *k* is related to the concentration at half-maximal response. The extracted parameters are shown in Table 1.

#### Electrophysiology

Cell-attached and outside-out patches were used as previously described^47^. All experiments were at room temperature. Pressure or suction steps were applied to the membranes using a high speed pressure clamp (ALA)^48^. The bath solution contained (in mM): 150 KCl, 10 HEPES, 1 MgCl_2_, 1 CaCl_2_ at pH 7.4 that clamped the resting membrane potential to zero. The pipette solution was 150 mM KCl at pH 7.4.

#### microRNAs

Two microRNAs targeting *PIEZO1* expression were cloned using the BLOCK-IT expression vector kit (Invitrogen) according to the manufacturer’s specifications. The following primers were used:

338_top TGCTGTAGACAATCTTGTAGACCACGGTTTTGGCCACTGACTGACCGTGGTCTAAGATTGTCTA

338_bot CCTGTAGACAATCTTAGACCACGGTCAGTCAGTGGCCAAAACCGTGGTCTACAAGATTGTCTAC

3767_top TGCTGTGGAACAGGTACTTGACGACCGTTTTGGCCACTGACTGACGGTCGTCATACCTGTTCCA

3767_bot CCTGTGGAACAGGTATGACGACCGTCAGTCAGTGGCCAAAACGGTCGTCAAGTACCTGTTCCAC

The two miRNAs were introduced into one vector (chaining) according to the manufacturer’s specifications.

#### Cell Migration

Half of a glass coverslip was coated with catalyzed PDMS (polydimethylsiloxane) (9:1) and allowed to harden. Cells were cultured on the complete coverslip kept in tissue culture dishes. After achieving confluency, the PDMS was peeled from the glass. The location of the front edge of the confluent cells was monitored for 5 hr at 37 C° with 5% CO_2_-supplemented DMEM media containing 10% bovine serum. Test peptides were added to the culture media at the start of the 5 hr experiment.

To analyze the average migration speed cell front, images were processed using Cell Profiler (open source software, http://cellprofiler.org/) to measure the area covered by the cells in each frame. Dividing by the width of the frame, an average (with SEM) speed of movement in a direction perpendicular to the cell front was calculated The datasets generated and/or analyzed during the current study are available from the corresponding author on request.

#### Super resolution microscopy

Samples were imaged on a Visitech VTI-iSIM attached to a Nikon TE2000 inverted microscope. Images of samples excited at 488 nm and 568 nm were captured at 60 z-intervals of 200 nm.

#### Cytoskeleton stress probe

To measure cytoskeletal stress, hP1-mCherry-CL cells were transiently transfected with cDNA encoding the cpst-actin FRET probe^49^. The FRET ratio of 528 nM and 475 nM was measured by simultaneous imaging using an image splitter. Baseline actin stress was measured at 37 °C in isotonic solution for 30 min before peptide addition (see above).

#### Transient transfection

Transient transfections were done at least 24 hours before experiments using Mirus T*rans*IT®−293 reagent according to manufacturer’s specification.

#### Peptide preparation

The enantiomeric forms of human Aβ(1-40) and Aβ(1-42) peptides were chemically synthesized by and purchased from Anaspec or Bachem. The scrambled Aβ(1-40) peptide, HiLyte (555) fluorescent peptide Aβ(1–42), and AggreSure peptide were purchased from Anaspec.

### Peptide preparation methods

#### Method I

To one mg of peptide, 70 μl 1.0% NH_4_OH was added. The peptide was then diluted with bath buffer (see above) to a concentration of approximately 1 mg/Ml, and gently vortexed and frozen at −80 °C. This method generated oligomeric peptide.

#### Method II

(monomer procedure^41^): One mg of peptide was dissolved in 0.5 ml TFA (trifluoro acetate) and sonicated for 10 min. TFA was removed with streaming argon after which 800 µl of 1,1,3,3,3-Hexafluoro-2-propanol (THIP) (Sigma-Aldrich) was added and incubated 1 hr at 37 °C. This solvent allows aggregated peptides to dissociate into monomers by interfering with interactions used to stabilize aggregate peptides. The solvent was removed by streaming argon, and 800 THIP was added and frozen to −80 °C. The material was lyophilized and dissolved in 5 mM DMSO (Sigma-Aldrich) and then diluted in buffer. All peptides were stored at −80 °C.

Peptide sequences were as follows:

Aβ(1-40) DAEFRHDSGYEVHHQKLVFFAEDVGSNKGAIIGLMVGVV

Aβ(1-42) DAEFRHDSGYEVHHQKLVFFAEDVGSNKGAIIGLMVGGVVIA

Aβ(1-40) Scrambled AEGDSHVLKEGAYMEIFDVQGHVFGGKIFRVVDLGSHNVA.

## Electronic supplementary material

Supplemental Dataset 1

## Data Availability

The datasets generated during and/or analyzed during the current study are available from the corresponding author on reasonable request.

## References

[1] Johnson VE, Stewart W, Smith DH (2010). Traumatic brain injury and amyloid-beta pathology: a link to Alzheimer’s disease?. Nat Rev Neurosci.

[2] Li J (2014). Piezo1 integration of vascular architecture with physiological force. Nature.

[3] Ranade SS (2014). Piezo1, a mechanically activated ion channel, is required for vascular development in mice. Proceedings of the National Academy of Sciences of the United States of America.

[4] Syeda R (2016). Piezo1 Channels Are Inherently Mechanosensitive. Cell Rep.

[5] Cox CD (2016). Removal of the mechanoprotective influence of the cytoskeleton reveals PIEZO1 is gated by bilayer tension. Nat Commun.

[6] Lewis, A. H. & Grandl, J. Mechanical sensitivity of Piezo1 ion channels can be tuned by cellular membrane tension. *Elife* **4**, 10.7554/eLife.12088 (2015). 10.7554/eLife.12088 PMC4718726 26646186

[7] Williams TL, Serpell LC (2011). Membrane and surface interactions of Alzheimer’s Abeta peptide–insights into the mechanism of cytotoxicity. FEBS J.

[8] Beaven AH (2017). Gramicidin A Channel Formation Induces Local Lipid Redistribution I: Experiment and Simulation. Biophysical Journal.

[9] Cox CD, Bavi N, Martinac B (2017). Origin of the Force: The Force-From-Lipids Principle Applied to Piezo Channels. Curr Top Membr.

[10] Bavi N (2016). The role of MscL amphipathic N terminus indicates a blueprint for bilayer-mediated gating of mechanosensitive channels. Nat Commun.

[11] Lundbaek JA, Koeppe RE, Andersen OS (2010). Amphiphile regulation of ion channel function by changes in the bilayer spring constant. Proceedings of the National Academy of Sciences of the United States of America.

[12] Guo, Y. R. & MacKinnon, R. Structure-based membrane dome mechanism for Piezo mechanosensitivity. *Elife* **6**, 10.7554/eLife.33660 (2017). 10.7554/eLife.33660 PMC5788504 29231809

[13] Pathak MM (2014). Stretch-activated ion channel Piezo1 directs lineage choice in human neural stem cells. Proceedings of the National Academy of Sciences of the United States of America.

[14] Eisenhoffer GT (2012). Crowding induces live cell extrusion to maintain homeostatic cell numbers in epithelia. Nature.

[15] Gudipaty SA (2017). Mechanical stretch triggers rapid epithelial cell division through Piezo1. Nature.

[16] Koser DE (2016). Mechanosensing is critical for axon growth in the developing brain. Nat Neurosci.

[17] Blumenthal NR, Hermanson O, Heimrich B, Shastri VP (2014). Stochastic nanoroughness modulates neuron-astrocyte interactions and function via mechanosensing cation channels. Proceedings of the National Academy of Sciences of the United States of America.

[18] Bae C, Sachs F, Gottlieb PA (2011). The mechanosensitive ion channel Piezo1 is inhibited by the peptide GsMTx4. Biochemistry.

[19] Guo J, Wang Y, Sachs F, Meng F (2014). Actin stress in cell reprogramming. Proceedings of the National Academy of Sciences of the United States of America.

[20] Maneshi MM, Sachs F, Hua SZ (2015). A threshold shear force for calcium influx in an astrocyte model of traumatic brain injury. Journal of neurotrauma.

[21] Gottlieb PA, Bae C, Sachs F (2012). Gating the mechanical channel Piezo1: a comparison between whole-cell and patch recording. Channels (Austin).

[22] Suchyna TM (2004). Bilayer-dependent inhibition of mechanosensitive channels by neuroactive peptide enantiomers. Nature.

[23] Nishizawa K (2015). Effects of Lys to Glu mutations in GsMTx4 on membrane binding, peptide orientation, and self-association propensity, as analyzed by molecular dynamics simulations. Biochimica et Biophysica Acta (BBA)-Biomembranes.

[24] Gnanasambandam R, Nishizawa K, Sachs F, Suchyna T (2013). Positively Charged Residues on GsMTx4 are Crucial for Inhibition of the Mechanosensitive Ion Channel Piezo1. Biophysical Journal.

[25] Bae C, Gnanasambandam R, Nicolai C, Sachs F, Gottlieb PA (2013). Xerocytosis is caused by mutations that alter the kinetics of the mechanosensitive channel PIEZO1. Proceedings of the National Academy of Sciences of the United States of America.

[26] Poole K, Herget R, Lapatsina L, Ngo HD, Lewin GR (2014). Tuning Piezo ion channels to detect molecular-scale movements relevant for fine touch. Nat Commun.

[27] Sachs F (2018). Mechanical Transduction and the Dark Energy of Biology. Biophysical Journal.

[28] Suchyna TM, Markin VS, Sachs F (2009). Biophysics and structure of the patch and the gigaseal. Biophys J.

[29] Qi Y (2015). Membrane stiffening by STOML3 facilitates mechanosensation in sensory neurons. Nat Commun.

[30] Anishkin A, Kung C (2013). Stiffened lipid platforms at molecular force foci. Proceedings of the National Academy of Sciences of the United States of America.

[31] Markin VS, Sachs F (2015). Free Volume in Membranes: Viscosity or Tension?. Open Journal of Biophysics.

[32] Kakio A, Nishimoto S, Yanagisawa K, Kozutsumi Y, Matsuzaki K (2002). Interactions of amyloid beta-protein with various gangliosides in raft-like membranes: importance of GM1 ganglioside-bound form as an endogenous seed for Alzheimer amyloid. Biochemistry.

[33] Kawarabayashi T (2004). Dimeric amyloid beta protein rapidly accumulates in lipid rafts followed by apolipoprotein E and phosphorylated tau accumulation in the Tg2576 mouse model of Alzheimer’s disease. J Neurosci.

[34] Nourse JL, Pathak MM (2017). How cells channel their stress: Interplay between Piezo1 and the cytoskeleton. Semin Cell Dev Biol.

[35] McHugh BJ (2010). Integrin activation by Fam38A uses a novel mechanism of R-Ras targeting to the endoplasmic reticulum. J Cell Sci.

[36] Nelson R, Eisenberg D (2006). Recent atomic models of amyloid fibril structure. Curr Opin Struct Biol.

[37] Selkoe DJ, Hardy J (2016). The amyloid hypothesis of Alzheimer’s disease at 25 years. EMBO Mol Med.

[38] Meng, F. *et al*. Highly disordered amyloid-β monomer probed by single-molecule FRET and MD simulation. *Biophys J in press* (2018). 10.1016/j.bpj.2017.12.025 PMC5984999 29490247

[39] Pearson HA, Peers C (2006). Physiological roles for amyloid beta peptides. J Physiol.

[40] Parihar MS, Brewer GJ (2010). Amyloid-beta as a modulator of synaptic plasticity. J Alzheimers Dis.

[41] Giuffrida ML (2009). Beta-amyloid monomers are neuroprotective. J Neurosci.

[42] Friedl P, Gilmour D (2009). Collective cell migration in morphogenesis, regeneration and cancer. Nat Rev Mol Cell Biol.

[43] McHugh BJ, Murdoch A, Haslett C, Sethi T (2012). Loss of the integrin-activating transmembrane protein Fam38A (Piezo1) promotes a switch to a reduced integrin-dependent mode of cell migration. PLoS One.

[44] Hijmans W, Sipe JD (1979). Levels of the serum amyloid A protein (SAA) in normal persons of different age groups. Clin Exp Immunol.

[45] Maneshi MM, Gottlieb PA, Hua SZ (2017). A Microfluidic Approach for Studying Piezo Channels. Curr Top Membr.

[46] Bae C, Sachs F, Gottlieb PA (2015). Protonation of the human PIEZO1 ion channel stabilizes inactivation. The Journal of biological chemistry.

[47] Gnanasambandam R, Bae C, Gottlieb PA, Sachs F (2015). Ionic Selectivity and Permeation Properties of Human PIEZO1 Channels. PLoS One.

[48] Besch SR, Suchyna T, Sachs F (2002). High-speed pressure clamp. Pflugers Arch.

[49] Meng F, Sachs F (2012). Orientation-based FRET sensor for real-time imaging of cellular forces. J Cell Sci.

