# Supplementary material for: Enantiomeric Aβ peptides inhibit the fluid shear stress response of PIEZO1
Source: Sci Rep. 2018 Sep 24;8:14267. doi: 10.1038/s41598-018-32572-2 (PMC6155315; doi:10.1038/s41598-018-32572-2)
Supplement: Supplementary file 1 — Supplemental Dataset 1 [file 41598_2018_32572_MOESM1_ESM.docx]

Enantiomeric Aβ peptides inhibit the fluid shear stress response of PIEZO1

Mohammad M. Maneshi, Lynn Ziegler, Frederick Sachs, Susan Z. Hua and Philip A. Gottlieb

-20 mV

-40 mV

-60 mV

-80 mV

-100 mV

2 pA

200 ms

**Supplemental Figure 1.** hPIEZO1-1591-GFP single channel currents. **Left** –Typical currents at five holding potentials in hP1-CL1 cells. The properties of these currents are nearly identical to those of transiently transfected wild type hPIEZO1 channels. The 500 ms suction pulse indicated above the traces applied a negative pressure of -60 mmHg. Right- The IV relationship for hP1-CL1. Single channel conductance from 3 patches at the indicated voltages were averaged (SD) and plotted (R^2^ = 0.99). The conductance is 42 pS at -100 mV with a reversal potential near 0 and is similar to the wild type channel^45^.


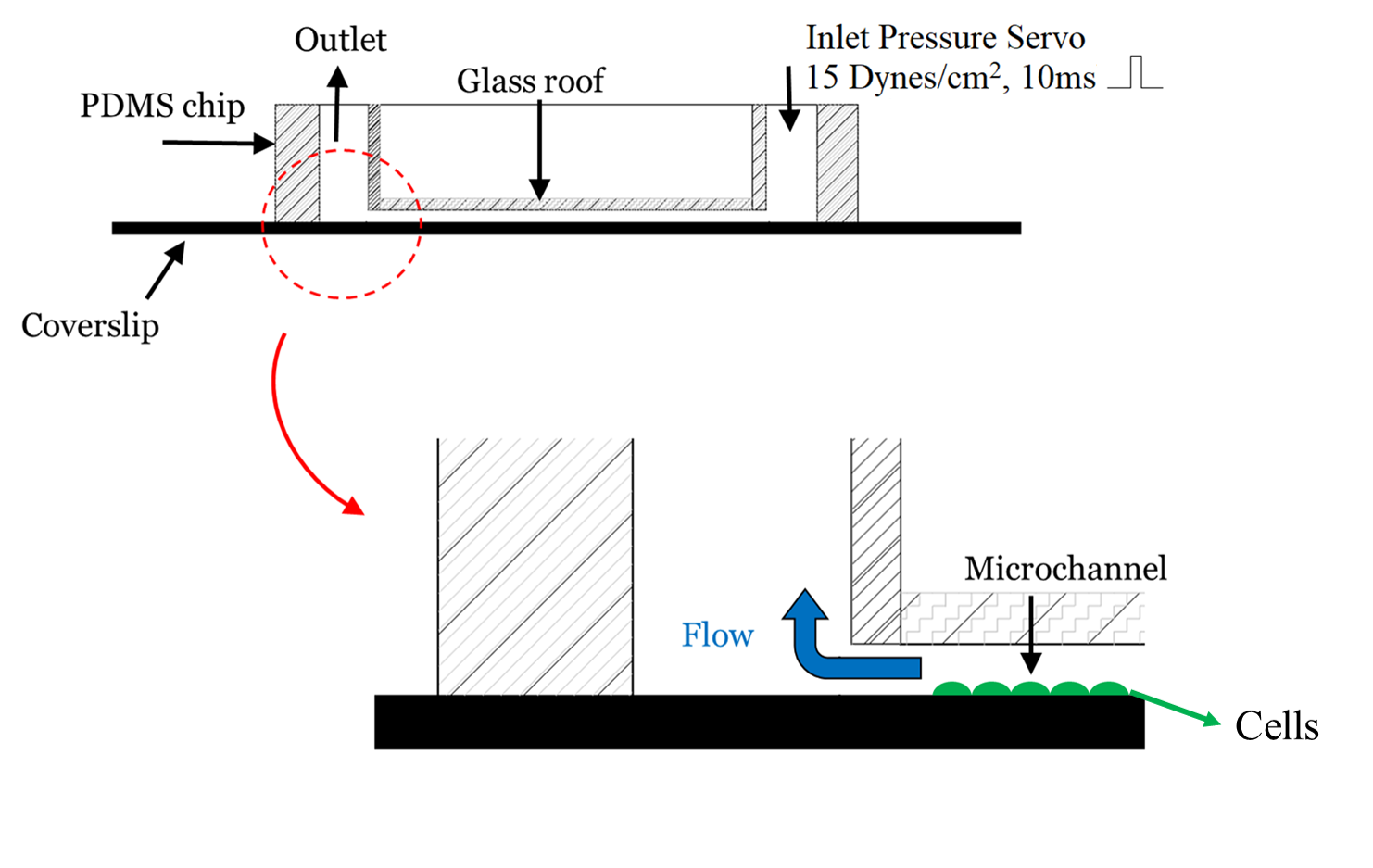


**Supplemental Figure 2**. Cross section of the chamber used to measure fluid shear stress. **Top Panel** shows the overall features of the chamber. The inlet is connected to a pressure servo and is used to deliver a single square pulse (indicated above the inlet). The glass roof forms the top of the chamber and the cover slip forms the bottom. **Bottom panel**-Expanded view of the area of the red circle in the top panel. Cells grown on the cover slip as part of the microchannel are indicated in green. The flow from the stimulus is shown as a blue arrow.

Inlet Pressure Servo

10 msec, 15 dynes/cm^2^


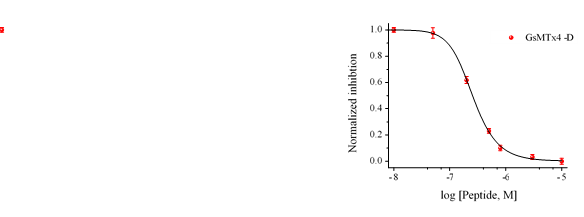

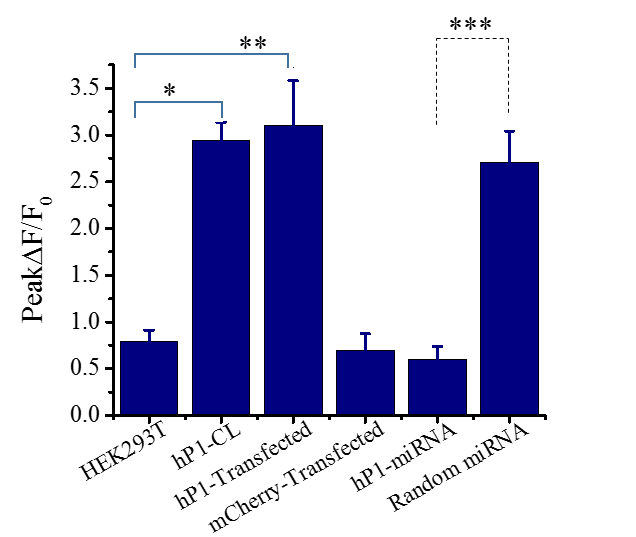

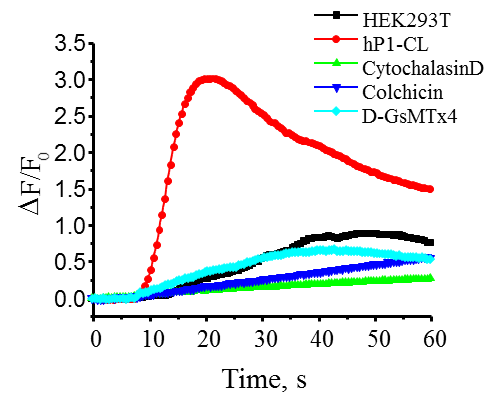


A B C

**Supplemental Figure 3. Panel A)** The time-dependent [Ca^2+^]_i_ response of hP1-CL cells(red circles) to a 10 ms shear pulse of 15 dynes/cm^2^ (marked by black arrow), over conditions as indicated (see key). Control cells (HEK293T) show a much smaller response with different kinetics (black). Treatment of the hP1-CL cells with either Cytochalasin D (blue triangles) or Colchicine (green triangles) abrogates the shear-induced Ca^2+^ response, implying that the cytoskeleton is involved in the shear activation by PIEZO1. Application of 5µM GsMTx4 (blue diamonds also inhibited the shear stress response of hP1-CL cells (standard error of the mean of 6 independent experiments). **Panel B**) The peak response of hP1-CL cells is significantly greater than that of control HEK293T cells (paired t-test * P<0.0001). Transient transfection of PIEZO1 (hP1-Transfected) produced the same Ca^2+^ response as hP1-CL cell line stably overexpressing hPIEZO1 (paired t-test **, P<0.0001). As a control, expression of mCherry alone had no effect on the response. MicroRNAs targeting PIEZO1 inhibited the response after two days but random microRNA sequences had no effect (paired t-test*** P<0.0001). All experiments were averaged for 4 independent experiments with SEM. **Panel C)** GsMTx4 titration of hP1-CL cells revealed a K_i_ for PIEZO1 of ~250 nM, similar to values previously reported^25^. GsMTx4 is bound with positive cooperativity of 1.9 (The errors are SEM for 3 independent experiment).

0

10

20

30

40

50

60

0.0

0.5

1.0

1.5

2.0

2.5

3.0

3.5

Time, s

Δ

F/F

0

HEK293T

hP1-CL

CytochalasinD

Colchicin

D-GsMTx4

Scrambled Aβ(1-40)

APP-YFP

HEK293T

hP1-EGFP-CL

hP1-EGFP-CL/APP

Scrambled Aβ(1-40)

0.0

0.5

1.0

1.5

2.0

2.5

3.0

3.5

Δ

F/F_0_

**Supplementary Figure 4.** Neither scrambled L-Aβ(1-40) nor Amyloid Precursor protein (APP) inhibit hPIEZO1 channels. ***Left panel*** - 10 µM scrambled L-Aβ(1-40) applied to hP1-CL cells (black arrow) (4 independent experiments with standard error of the mean). ***Middle panel*** - Heterologous expression in hP1-CL cells of Amyloid Precursor protein (APP) linked C-terminally to yellow fluorescent protein YFP did not affect the cell response to applied fluid shear stress (black arrow) (4 independent experiments with standard error of the mean). ***Right panel*** - Comparison of peak [Ca^2+^]_i_ responses of the indicated cell lines and test conditions to shear stress. This summarizes the lack of PIEZO1 inhibition by APP expression or by exogenous scrambled L-Aβ(1-40).


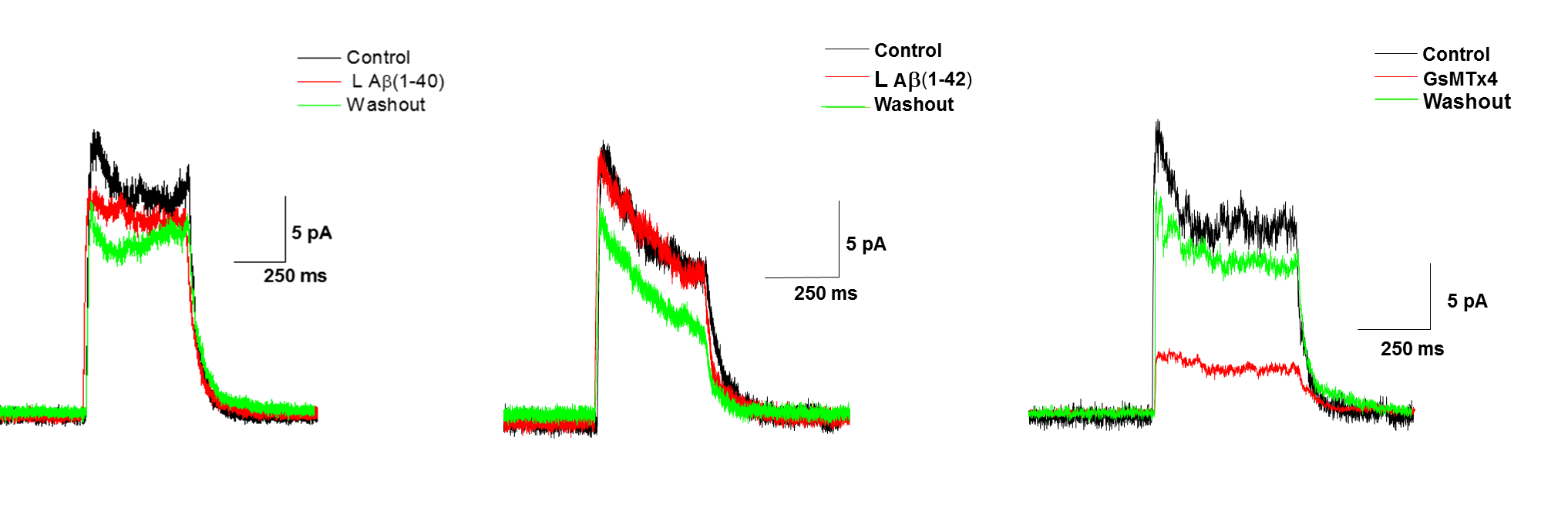


**Supplemental Figure 5**. Aβ peptides do not inhibit PIEZO1 channels in Outside/Out (O/O) patches (3 patches were tested). Shown above are typical results from single patches. **Left-** a positive pressure pulse of 60 mmHg at 50 mV lasting 500 msec was applied to an O/O patch every 2 seconds. After 12 pulses (24 seconds, black trace is the average of 12 pulses), 10 µM of L Aβ(1-40) was applied by perfusion and activity was monitored for 30 seconds (red trace is the average of 15 pulses). No significant reduction in current was observed. Washout also had minimal effect on channel activity (green trace is an average of 12 pulses). **Center-** The effect of Aβ(1-42) on a different patch (identical conditions described above). 12 pressure pulses (black trace) established a baseline response. Application of Aβ(1-42) did not diminish the PIEZO1 response (average of 10 pulses-red trace) and washout had a minimal effect on activity. **Right-** GsMTx4 inhibits PIEZO1. 6 pressure pulses (50 mV, 60 mmHg) were applied to establish the baseline response (black). GsMTx4 at 5 µM was perfused for 10 pressure pulses (red) followed by washout of the peptide with 10 pressure pulses (green). Inhibition was 0.67 ± 0.07 for 3 independent experiments.
